# Subjective persistent dizziness handicap and psychological factors in vestibular schwannoma patients: a cross-sectional study

**DOI:** 10.1038/s41598-026-64384-0

**Published:** 2026-07-29

**Authors:** M. Rutenkröger, M. Scheer, S. Rampp, C. Strauss, R. Schönfeld, B. Leplow

**Affiliations:** 1 Department of Medical Psychology, University Medical Center Hamburg- Eppendorf, Hamburg, Germany; 2 Department of Neurosurgery, University Hospital Halle, Halle, Germany; 3 Department of Neurosurgery, University Hospital Heidelberg, Heidelberg, Germany; 4 Department of Neurosurgery, University Hospital Erlangen, Erlangen, Germany; 5 Department of Neuroradiology, University Hospital Erlangen, Erlangen, Germany; 6 Department of Psychology, Martin-Luther-Universität Halle-Wittenberg, Halle, Germany

**Keywords:** Vestibular schwannoma, Dizziness, Vertigo, Microsurgery, Personality, Psychological factors, Human behaviour, Quality of life

## Abstract

Persistent dizziness is a frequent and disabling symptom after retrosigmoid microsurgery for vestibular schwannoma (VS), negatively affecting quality of life. While vestibular outcomes are well studied, the contribution of psychological factors to persistent dizziness remains insufficiently understood. This cross-sectional study included 93 patients with VS, of whom 80.6% reported postoperative dizziness. Psychological factors were assessed using validated self-report questionnaires covering premorbid mental health conditions, personality traits, somatization, anxiety, depression, and psychological distress. Dizziness-related impairment was quantified using the Dizziness Handicap Inventory (DHI). Associations were examined using Spearman correlations with bootstrap resampling, followed by multivariable linear regression to identify independent predictors of dizziness handicap. Dizziness handicap was robustly associated with vestibular symptom burden, somatization, anxiety, and psychological distress. Higher anxiety and somatization scores were consistently related to greater dizziness-related impairment, particularly in emotional and functional domains. Higher scores on the GBI Social Support subscale, reflecting surgery-related changes in the need for and reliance on social support rather than baseline social support resources, were associated with greater dizziness handicap and independently predicted higher DHI scores. Personality traits showed heterogeneous associations; conscientiousness was positively associated with physical dizziness handicap and showed a trend-level association with overall handicap, potentially reflecting increased symptom monitoring or health awareness. Given the cross-sectional design and absence of objective vestibular testing, causal relationships cannot be established. Persistent dizziness after VS surgery likely reflects multifactorial mechanisms involving vestibular, psychological, and functional contributors. Future longitudinal studies integrating comprehensive neuro-otological assessments are needed to clarify temporal relationships and underlying mechanisms.

## Introduction

A vestibular schwannoma (VS), also referred to as an acoustic neuroma, is a benign neoplasm that arises from the Schwann cells of the vestibulocochlear nerve. While the primary consequence of this condition is often auditory, many patients also present with dizziness and vertigo, which may persist after or be induced by microsurgery or radiation treatment^1–3^. Conservative VS management (“wait-and-scan”) seems to have no impact on the prevalence of these symptoms, as well as postural sway and canal paresis^4^. The severity of these vestibular symptoms is variable, but they frequently have a long-lasting impact on patients’ quality of life (QoL) across multiple domains^2,3,5^.

As identified by Carlson et al.^5^, the strongest predictors of long-term QoL reduction in patients with sporadic VS are ongoing dizziness and headache. In comparison, the impact of hearing loss, facial nerve function, and tinnitus was shown to be less significant^5^. Dizziness is significantly correlated with challenges in performing routine tasks, which can lead to a deterioration in physical well-being, an increased reliance on external assistance, and occupational limitations^6^. It has been demonstrated that dizziness is linked to elevated levels of anxiety, depression, and diminished cognitive function, which serves to further exacerbate the decline in QoL^6,7^. Furthermore, balance disorders were found to be associated with anxiety and depression, as well as with greater daily consequences and a denial coping response^8^. In an experimental setting, the presence of anxiety symptoms during a vestibular stimulus may contribute to a priming effect that could explain the observed worsening of balance function^9^.

In addition to the dizziness associated with VS, it has also been shown that somatoform complaints in individuals experiencing dizziness are highly prevalent. The presence of certain factors, such as personality characteristics or accompanying psychopathology, has been identified as influencing the prevalence of these complaints^10^. Research on chronic subjective dizziness^11^ and persistent postural-perceptual dizziness (PPPD)^12^ has revealed that anxiety, neuroticism, and somatic symptom burden are markedly elevated in individuals with these conditions. Patients with PPPD also exhibited diminished levels of conscientiousness in comparison to the control group^12^. In the aftermath of acute vestibular events, psychological and behavioral responses, along with brain maladaptation, were shown to be the most probable predictors of PPPD^13^. Furthermore, a correlation was identified between benign paroxysmal positional vertigo and the presence of comorbid major depressive disorder, generalized anxiety disorder, and obsessive-compulsive personality disorders when compared to the findings in a control group^14^.

Given the lack of comprehensive examination of psychological factors in patients with VS, the objective of this study was to evaluate the associations between psychological, clinical, and sociodemographic factors and postoperative dizziness-related handicap following retrosigmoid microsurgery. Specifically, the present study aimed to examine, across the full cohort, the relationships between dizziness handicap and premorbid psychological conditions, personality traits, somatization tendencies, current symptoms of depression and anxiety, perceived postoperative health benefit and social support, as well as sociodemographic and clinical variables, including age, gender, tumor size, and time since treatment. In addition, a multivariable regression analysis was performed to identify factors independently associated with postoperative dizziness handicap while accounting for potential confounding between psychological and clinical variables.

## Materials and methods

This cross-sectional, single-center investigation was conducted at University Hospital Halle (Saale), Germany, as part of a broader study that also explored psychological factors and their associations with postoperative headache and tinnitus in patients with VS patients^15^. The survey was initiated and conducted to derive relevant data directly from healthcare provision, which is why no additional neuro-otological examinations were performed. The inclusion criteria were as follows: individuals with a confirmed diagnosis of VS, aged 18 or above at the time of diagnosis, undergoing retrosigmoid surgery, and possessing proficiency in the German language. Patients were excluded if they had undergone prior surgical procedures or radiation therapy, if they had a history of recurrent VS, if they had been diagnosed with additional oncological conditions, or if they had been diagnosed with neurofibromatosis type 2. The advent of the SARS-CoV-2 pandemic precipitated a postponement in the recruitment of subjects, with the study commencing in early 2020. The initial stage of participant engagement was conducted through direct approaches during resident consultations at University Hospital Halle. Subsequently, an online survey comprising identical questions was administered by *Vereinigung Akustikus Neurinom e.V.* (a non-profit patient self-help organization) following the national lockdown due to the spread of the virus (SoSciSurvey). Prior to participation, all subjects provided written informed consent. The study was conducted in accordance with the Declaration of Helsinki, and the protocol was approved by the Ethics Committee of University Hospital Halle (No. 2020-008).

### Measures

A self-administered survey was employed to collect data pertaining to demographic characteristics (e.g., age and gender), psychological aspects (including premorbid psychological diagnoses such as anxiety disorders or other mental conditions such as sleep disturbances), and treatment details (e.g., surgical approach, craniotomy vs. craniectomy). The survey consisted of binary questions (yes/no), with the option of providing additional details when necessary (e.g., “Specify the diagnosed psychological disorder”). Tumor size was evaluated according to the Koos grading system, which ranges from 1 to 4^16^. Severity of preoperative symptoms such as dizziness, tinnitus, headache, and hearing loss were retrospectively rated on a numeric analog scale (NAS) of 1–10. Severity of postoperative dizziness was also assessed on a NAS ranging from 1 to 10.

The German version of the Dizziness Handicap Inventory, known as DHI-G^17^, was employed for the purpose of evaluating the extent of disability associated with dizziness. The scale comprises 25 items, with a score of 4 allocated to affirmative responses, 2 to “sometimes” responses, and 0 to negative responses. The total score ranges from 0, indicating no disability, to 100, reflecting severe disability. The questionnaire is comprised of three subscales: a seven-item physical subscale, a nine-item emotional subscale, and a nine-item functional subscale. The DHI-G has been demonstrated to perform reliably, and it is therefore recommended as a suitable assessment tool for gauging disability in individuals experiencing dizziness and unsteadiness.

The Glasgow Benefit Inventory (GBI) is a validated questionnaire comprising 18 items that is utilized to assess patients’ perceived health benefits (PHB) post-intervention, such as surgery. GBI scores range from − 100 to + 100, with a score of 0 indicating no perceived benefit, + 100 signifying the highest level of benefit, and negative scores suggesting a decline in health. Principal component analysis revealed that the Glasgow Benefit Inventory items consistently fell into three distinct subscales. The first subscale, labeled “General,” comprised twelve questions related to overall health changes, both general and psychosocial. The second subscale, labeled “Social,” included three questions addressing the necessary social support for the specific condition at hand. The final subscale, labeled “Physical,” included three questions focusing on changes in physical health status, including medication requirements and doctor visits^18^.

In order to assess the Big Five personality traits in accordance with the framework established by McCrae and Costa^19^, the German version of the Ten Item Personality Inventory (TIPI-G)^20^ was utilized. This inventory captures the five fundamental dimensions of the personality model, namely emotional stability (the opposite of neuroticism), extraversion, agreeableness, openness, and conscientiousness. The TIPI-G, which was designed as a concise version of the questionnaire, is particularly beneficial in situations with time constraints, as it provides a reliable estimate of the comprehensive measurements associated with the five-factor model of personality, such as those provided by the NEO-PI-R^21^. The respondents employ a 7-point Likert scale (ranging from 1 for “strongly disagree” to 7 for “strongly agree”) to evaluate ten pairs of adjectives. The resulting scale score is the mean of two items, including one negatively worded item.

Premorbid somatization tendencies were assessed using the Screening for Somatoform Disorders (SOMS-2), a validated instrument commonly used in patient populations with psychosomatic disorders^22^. In the first part of the questionnaire, participants were asked to report physical symptoms that were intermittent or persistent in the two years prior to the diagnosis of VS. These symptoms should have significantly interfered with their well-being or personal lifestyle, without a clearly identified cause as determined by medical professionals. The questionnaire presented a list of 53 somatoform symptoms, 5 specific to women and 1 specific to men, based on criteria from the Diagnostic and Statistical Manual of Mental Disorders (DSM-IV-TR) and the International Classification of Diseases (ICD-10). The second section consisted of 15 questions designed to assess disability, frequency of medical consultations due to symptoms, and inclusion/exclusion criteria for all somatoform disorders.

We used the Hospital Anxiety and Depression Scale (HADS-D) to assess current symptoms of anxiety and depression^23^. This questionnaire consists of two scales, one for anxiety and the other for depression, each ranging from 0 to 21. A total score, ranging from 0 to 42, was calculated based on symptoms experienced in the week prior to the assessment. Elevated scores indicated increased levels of anxiety, depression, or general psychological distress. The HADS-D serves as a screening tool, with scores greater than 8 warranting additional assessment for an affective disorder.

The statistical analysis was conducted using the statistical software package SPSS^24^, with a significance level set at α = 0.05. Data distribution was assessed using the Shapiro–Wilk test, which indicated non-normality of the variables. Accordingly, non-parametric methods were applied. Associations between study variables were examined using Spearman’s rank-order correlation coefficients (rs). Given the exploratory nature of the correlation analyses and the substantial conceptual and statistical interdependence among the DHI subscales and psychological measures, no formal adjustment for multiple comparisons was applied. The analyses were intended to identify patterns of association rather than to test a set of independent confirmatory hypotheses. To reduce reliance on asymptotic significance testing, associations were evaluated using bootstrap-derived 95% confidence intervals based on 1,000 resamples. Correlations were considered robust when the corresponding 95% bootstrap confidence interval did not include zero. Accordingly, significance markers and the textual interpretation of the results were aligned with the bootstrap confidence intervals. In addition, a multiple linear regression analysis was conducted to identify independent predictors of postoperative dizziness severity (DHI score). Predictor variables included gender, time since treatment, depressive and anxiety symptoms (HADS), somatization (DSM-IV index), perceived social support (GBI), and personality traits (TIPI conscientiousness). Multicollinearity was assessed using tolerance and variance inflation factors (VIF). All tests were two-tailed.− 

## Results

### Sample characteristics

A total of 93 participants were included in the final analysis, following the exclusion of eight individuals whose questionnaires were incomplete or lacked data regarding tumor size. Of the evaluated participants, 75 (80.6%) reported experiencing postoperative dizziness. It is noteworthy that over half of the patients surveyed indicated that they had also experienced dizziness prior to surgery. The participants’ ages ranged from 17 to 72 years, with a mean age at treatment of 47.5 years (SD = 11.1) and the mean duration since treatment was 9.1 years (range 3 months to 36 years). The preoperative dizziness severity scores, as measured by the NAS, ranged from 1 to 10, with a mean score of 4.0 (SD = 2.6). Similarly, postoperative dizziness severity scores ranged from 1 to 10, with a mean score of 4.6 (SD = 2.8). Of the 75 patients who reported postoperative dizziness, a total of 19.4% of participants reported experiencing a severe degree of dizziness-related handicap as assessed by the DHI-G. All patients who reported preoperative dizziness also experienced postoperative dizziness, and 24 patients (25.8%) reported that dizziness began to occur after surgery. Table 1 presents the descriptive statistics for the additional demographic variables and questionnaire scales. The descriptive data for the administered questionnaires are presented in Table 2.

**Table 1:** Absolute and relative frequencies of demographic variables (*N* = 93).

Variable	Category	*N*	%
Gender	Female	56	60.2
	Male	37	39.8
Premeorbid psychiatric condition		18	19.4
Premorbid psychiatric symptoms		29	31.2
Preoperative vertigo/dizziness		51	54.8
Preoperative headache (baseline symptom)		30	32.3
Preoperative tinnitus/ear noise		50	53.8
Preoperative hearing loss		47	50.5
Dizziness severity (DHI-G) (N=75)	Mild	34	36.6
	Moderate	31	33.3
	Severe	10	19.4

**Table 2:** Descriptive data of used questionnaires (*N* = 93).

Scale	*N*	Min	Max	M (SD)
*DHI*				
Total	93	0	92	27.99 (22.84)
Physical	93	0	28	9.30 (7.74)
Emotional	93	0	28	8.15 (7.73)
Functional	93	0	36	10.54 (8.82)
*Tinnitus*				
TBF score	93	0	24	7.71 (6.26)
*Somatization*				
Symptom count	93	0	35	8.14 (6.62)
Somatization index DSM-IV	93	0	14	3.46 (3.19)
Somatization index ICD	93	0	22	2.63 (3.39)
*Global Benefit*				
GBI global	93	-100	75	− 19.58 (31.66)
GBI social support	93	-83	67	2.51 (23.31)
GBI physical health	93	-83	33	− 16.81 (27.77)
GBI overall	93	-81	64	− 16.02 (23.34)
*TIPI*				
Extraversion	93	2	14	8.29 (3.05)
Agreeableness	93	6	14	10.57 (2.11)
Conscientiousness	93	8	14	12.26 (1.60)
Emotional stability	93	2	14	9.94 (2.99)
Openness	93	4	14	10.43 (2.49)
*HADS-D*				
Depression	93	0	21	5.00 (3.32)
Anxiety	93	0	18	7.11 (3.87)
Total	93	2	39	12.11 (6.45)

DHI-G = Dizziness Handicap Inventory, TIPI-G = Ten Item Personality Inventory-German; GBI = Glasgow Benefit Inventory; SOMS-2 = Screening for Somatoform Disorders; HADS-D = Hospital Anxiety and Depression Scale.

#### Triggers for dizziness

**Fig. 1:**
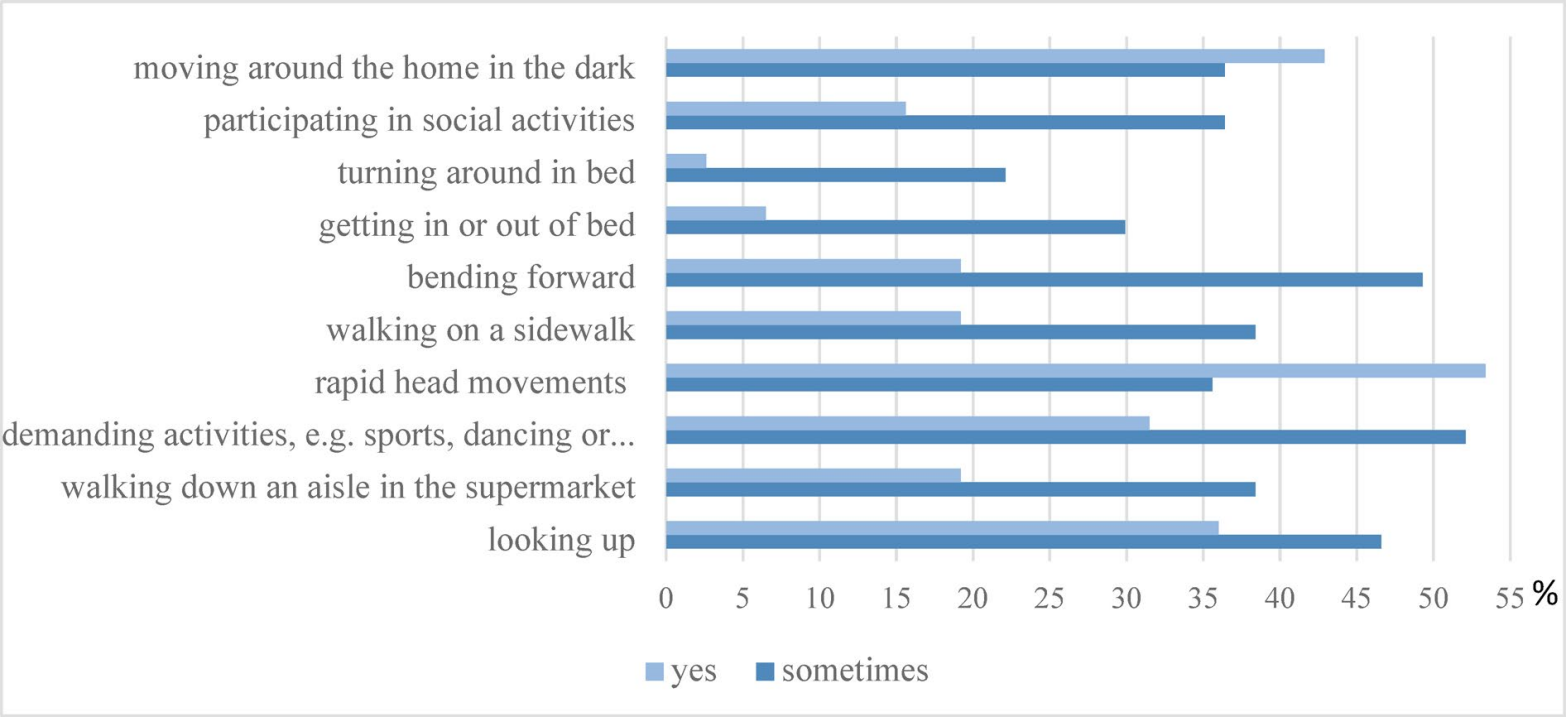
Triggers of dizziness as reported in the DHI-G (*n* = 73).

Of the 75 patients who reported dizziness, *n* = 2 reported that the dizziness occurred without an obvious trigger. An overview of the triggers reported by the remaining 73 patients is shown in Fig. 1.

A total of 20 patients reported a combination of the following triggers: activities such as looking up, walking down an aisle in the supermarket, engaging in demanding tasks like dancing, rapid head movements, walking on a sidewalk, and bending forward exacerbated their dizziness (scores of 2 = “sometimes” or 4 = “yes”). Additionally, 14 patients experienced dizziness exacerbation when getting in or out of bed and turning around in bed, suggesting symptoms consistent with vestibular dizziness, potentially indicating positional or peripheral vestibular dysfunction. Furthermore, 34 patients reported increased dizziness during social activities and while moving around the home in the dark, highlighting challenges related to visual dependency and sensory integration.

### Associations between postoperative dizziness handicap and psychological factors

Spearman correlation analyses with bootstrap resampling (1,000 samples; 95% confidence intervals) were conducted to examine the associations between dizziness-related impairment and clinical and psychological variables.

Overall dizziness handicap (DHI total score) showed significant positive associations with tinnitus symptom burden (TBF: *r* = .43, *p* < .001), somatization (DSM-IV: *r* = .34, *p* < .001), emotional stability (*r* = − .26, *p* < .05),  anxiety (*r* = .46, *p* < .001), and overall psychological distress (HADS total: *r* = .42, *p* < .001). Conversely, higher perceived global treatment benefit (GBI total score: *r* = − .30, *p* < .01) and better emotional well-being (GBI general well-being: *r* = − .41, *p* < .01) were associated with lower dizziness-related impairment. Female gender was positively associated with higher DHI scores (*r* = .33, *p* < .01). Age, tumor size (Koos grade), and time since treatment were not significantly associated with DHI scores (all *p* > .05).

Subscale analyses revealed consistent patterns across dizziness-related impairment domains. The physical subscale (DHI-P) was positively associated with female gender (*r* = .38, *p* < .01), vestibular symptom burden (TBF: *r* = .27, *p* < .01), somatization (DSM-IV: *r* = .27, *p* < .01), postoperative changes in social support needs (GBI Social Support subscale: *r* = .33, *p* < .01), and anxiety (*r* = .37, *p* < .001). The emotional subscale (DHI-E) showed the strongest associations with vestibular symptom burden (*r* = .48, *p* < .001), somatization (*r* = .32, *p* < .01), global well-being (GBI: *r* = − .49, *p* < .001), emotional stability (*r* = − .29, *p* < .001), anxiety (*r* = .49, *p* < .001), depression (*r* = .24,* p*  < .05) and overall distress (*r* = .44, *p* < .001). Similarly, the functional subscale (DHI-F) was significantly associated with vestibular symptom burden (*r* = .45, *p* < .001), somatization (*r* = .36, *p* < .001), postoperative changes in social support needs (GBI Social Support subscale: *r* = .29, *p* < .01), emotional stability (*r* = − .23, *p* < .05), anxiety (*r* = .44, *p* < .001), and lower perceived treatment benefit (GBI total: *r* = − .31, *p* < .01). Bootstrap confidence intervals supported the robustness of the majority of observed associations.

A multiple linear regression (Tables 3 and 4) was performed to predict DHI scores from gender, time since treatment, depressive and anxiety symptoms (HADS), somatization (DSM-IV index), perceived social support (GBI), and conscientiousness (TIPI). The model was significant, *F*(7, 85) = 6.88, *p* < .001, explaining 36.2% of the variance (*R²* = 0.362, adj. *R²* = 0.309).

Significant predictors of higher DHI scores were female gender (β = 0.219, *p* = .021), higher anxiety (β = 0.323, *p* = .008), higher scores on the GBI Social Support subscale, reflecting a greater surgery-related change in the need for social support rather than higher baseline social support availability (β = 0.255, *p* = .005). Conscientiousness showed a trend-level association (β = 0.166, *p* = .079). Time since treatment, depressive symptoms, and somatization were not significant predictors (all *p* ≥ .20). Multicollinearity was low across predictors (VIF = 1.04–1.91).

**Table 3:** The HADS total score was excluded due to overlap with subscales.

Variable	DHI total ρ [95% CI]	DHI physical ρ [95% CI]	DHI emotional ρ [95% CI]	DHI functional ρ [95% CI]
Age at treatment	0.06 [− 0.16, 0.27]	0.05 [− 0.17, 0.26]	0.04 [− 0.17, 0.25]	0.09 [− 0.14, 0.30]
Gender	0.33** [0.13, 0.52]	0.38** [0.19, 0.55]	0.27** [0.07, 0.47]	0.32** [0.12, 0.50]
Koos grade	0.05 [− 0.15, 0.24]	0.03 [− 0.17, 0.23]	0.09 [− 0.11, 0.29]	0.01 [− 0.19, 0.21]
Time since treatment	− 0.06 [− 0.28, 0.16]	− 0.06 [− 0.28, 0.14]	− 0.08 [− 0.28, 0.13]	− 0.05 [− 0.27, 0.16]
TBF score	0.43** [0.23, 0.60]	0.27** [0.05, 0.46]	0.48** [0.30, 0.65]	0.45** [0.25, 0.62]
*Somatization*				
SOMS-2 somatic symptom index	0.29** [0.11, 0.47]	0.22* [0.02, 0.42]	0.28** [0.10, 0.46]	0.32** [0.13, 0.50]
SOMS-2 Somatization Index (DSM-IV)	0.34** [0.15, 0.53]	0.27** [0.08, 0.46]	0.32** [0.13, 0.51]	0.36** [0.16, 0.55]
SOMS-2 Somatization Index (ICD)	0.05 [− 0.14, 0.24]	− 0.02 [− 0.21, 0.18]	0.07 [− 0.12, 0.25]	0.08 [− 0.11, 0.28]
*Global benefit*				
GBI general well-being	− 0.41** [− 0.56, − 0.22]	− 0.27* [− 0.45, − 0.07]	− 0.49** [− 0.63, − 0.32]	− 0.40** [− 0.56, − 0.22]
GBI social Support	0.31** [0.14, 0.49]	0.33** [0.15, 0.50]	0.25* [0.07, 0.43]	0.29** [0.12, 0.46]
GBI physical health	− 0.12 [− 0.32, 0.08]	− 0.03 [− 0.23, 0.17]	− 0.17 [− 0.36, 0.03]	− 0.14 [− 0.33, 0.07]
GBI total score	− 0.30** [− 0.48, − 0.12]	− 0.14 [− 0.34, 0.05]	− 0.41** [− 0.57, − 0.21]	− 0.31** [− 0.50, − 0.13]
*Personality*				
TIPI extraversion	− 0.15 [− 0.34, 0.06]	− 0.13 [− 0.34, 0.08]	− 0.10 [− 0.28, 0.12]	− 0.13 [− 0.32, 0.08]
TIPI agreeableness	− 0.01 [− 0.22, 0.20]	0.04 [− 0.18, 0.26]	− 0.03 [− 0.23, 0.19]	− 0.01 [− 0.21, 0.20]
TIPI conscientiousness	0.19 [− 0.01, 0.37]	0.22* [0.03, 0.41]	0.12 [− 0.06, 0.32]	0.19 [− 0.01, 0.39]
TIPI emotional stability	− 0.26* [− 0.46, − 0.04]	− 0.23* [− 0.42, − 0.01]	− 0.29** [− 0.49, − 0.08]	− 0.21 [− 0.43, 0.00]
TIPI openness	− 0.21 [− 0.40, 0.01]	− 0.14 [− 0.34, 0.08]	− 0.21 [− 0.40, 0.01]	− 0.20 [− 0.39, 0.03]
*Psychological burden*				
HADS depression	0.21 [− 0.01, 0.40]	0.08 [− 0.12, 0.27]	0.24* [0.04, 0.43]	0.21 [− 0.01, 0.40]
HADS anxiety	0.46** [0.30, 0.62]	0.37** [0.19, 0.54]	0.49** [0.33, 0.64]	0.44** [0.28, 0.60]
HADS total	0.42** [0.24, 0.57]	0.31** [0.11, 0.48]	0.44** [0.28, 0.58]	0.41** [0.24, 0.56]

Values represent Spearman’s rho. Bootstrapping was performed with 1,000 resamples (bias-corrected 95% confidence intervals). * *p* < .05, ** *p* < .01 (two-tailed).

*Abbreviations*. DHI-G = Dizziness Handicap Inventory, TIPI-G = Ten Item Personality Inventory-German; GBI = Glasgow Benefit Inventory; SOMS-2 = Screening for Somatoform Disorders; HADS-D = Hospital Anxiety and Depression Scale.

**Table 4:** Multiple linear regression predicting DHI total score (*N* = 93).

Predictor	B	SE B	β	t	*p*	VIF
Constant	−31.67	18.52	—	−1.71	0.091	—
Gender	10.17	4.31	0.219	2.36	0.021	1.15
Time since treatment	−0.25	0.24	−0.093	−1.04	0.303	1.08
HADS depression	−0.14	0.76	−0.020	−0.18	0.857	1.65
HADS anxiety	1.90	0.71	0.323	2.70	0.008	1.91
Somatization (DSM-IV)	0.91	0.74	0.127	1.24	0.220	1.40
GBI social support	0.25	0.09	0.255	2.89	0.005	1.04
TIPI conscientiousness	2.37	1.33	0.166	1.78	0.079	1.16

## Discussion

To the best of our knowledge, this is the first study to examine the associations between persistent dizziness and several psychological factors, such as personality and somatization, in VS patients who underwent retrosigmoid microsurgery. An understanding of these relationships is essential for the development of more effective treatment strategies that address both the physical and psychological dimensions of dizziness in this patient population. The present data show that dizziness handicap was consistently associated with tinnitus, somatization, and psychological distress. However, given the cross-sectional design and absence of objective vestibular testing, these findings should be interpreted as associations rather than evidence for specific diagnostic entities. Although symptom patterns such as visually induced dizziness or motion sensitivity are compatible with PPPD, no clinical diagnostic confirmation was available. Therefore, PPPD can only be considered a plausible explanatory framework rather than a confirmed diagnosis^25^

Sex differences observed in the present study, with female gender emerging as an independent predictor of higher dizziness handicap in the regression analysis, are consistent with previous findings in VS. Machetanz et al.^26^ reported that female VS patients experience greater dizziness, headache, and anxiety burden in both pre- and postoperative quality of life assessments compared to male patients, despite comparable or even greater overall postoperative improvement. These results suggest sex-related differences in symptom perception and disability reporting in VS. Our findings extend this evidence by showing that gender remains an independent predictor of dizziness handicap even after controlling for psychological factors such as anxiety, somatization, and perceived social support, indicating that sex-related effects are not fully explained by psychological distress alone.

Retrospective reports of preoperative dizziness among some patients introduce an important consideration: vestibular dysfunction predating surgery may contribute to chronic dizziness, particularly when combined with the vestibular insult caused by microsurgery. Despite this, previous research has demonstrated that preoperative vestibular symptoms are predictive of postoperative dizziness^1–3^. Nevertheless, the substantial heterogeneity in time since treatment (3 months to 36 years) and reliance on retrospective symptom recall introduces uncertainty and potential recall bias. Additionally, there was considerable variability in the time since treatment, which may influence symptom persistence and compensation processes. The role of central vestibular compensation is also relevant, particularly in cases with persistent or decompensated symptoms. Other contributing mechanisms, such as bilateral vestibular dysfunction, vestibular migraine, visual or somatosensory deficits, and age-related vestibular decline, may also play a role.

Psychological factors appear to be associated with dizziness severity and may contribute to symptom persistence. Higher anxiety and somatization scores were consistently related to greater emotional and functional disability, in line with previous findings in chronic vestibular disorders^27,28^. Depression showed weaker associations, with bootstrap confidence intervals excluding zero only for the emotional subscale, suggesting that anxiety-related mechanisms may be more consistently linked to dizziness severity in this cohort^29^. Importantly, anxiety, depression, and somatization are strongly interrelated constructs; therefore, bivariate correlations may partially reflect shared variance rather than independent effects. Personality traits showed more heterogeneous associations. Conscientiousness demonstrated positive associations with dizziness handicap, suggesting that higher conscientiousness may reflect increased symptom monitoring or heightened health awareness rather than a protective effect. This finding contrasts with common assumptions regarding adaptive personality traits and underscores the complexity of personality–symptom relationships in chronic conditions^30^.

The observed correlation between somatization and the emotional aspects of dizziness further highlights the role of psychological burden in vestibular symptomatology, aligning with findings that somatic symptom disorder is prevalent in patients with vertigo and dizziness symptoms^31^.

Higher scores on the GBI Social Support subscale were associated with greater dizziness-related impairment and independently predicted higher DHI scores in the multivariable analysis. Importantly, this finding should not be interpreted as indicating that greater baseline social support increases dizziness handicap. The GBI assesses perceived change following an intervention, and its Social Support subscale reflects surgery-related changes in the need for and receipt of support from family and friends. Thus, the positive association may indicate that patients experiencing greater dizziness-related disability may experience greater changes in their need for or reliance on social support after surgery. This interpretation is consistent with the cross-sectional nature of the data and does not imply a causal effect of social support on dizziness severity. In contrast, higher scores on the GBI General Well-being and Total Score were associated with lower dizziness handicap in the bivariate analyses, suggesting that a more favorable perceived postoperative health outcome was related to lower symptom burden.

Taken together, the results indicate that persistent dizziness after microsurgery is likely multifactorial, involving interactions between residual vestibular dysfunction, central processing mechanisms, and psychological factors. Surgery addresses the tumor but does not necessarily resolve pre-existing vestibular vulnerability or prevent functional symptom persistence.

Multidisciplinary management approaches should be explored to address this issue. Combining vestibular rehabilitation with cognitive-behavioral therapy (CBT) or other psychological interventions may represent a promising approach for patients with persistent dizziness, particularly those with psychological comorbidities. For example, education about altered sensory processing, combined with strategies to improve postural control and reduce maladaptive behavioral responses, as well as relaxation techniques could help patients regain confidence in managing their symptoms^32^. While complete symptom resolution may not always be achievable, such targeted interventions offer the potential for significant improvements in symptom burden and QoL^33,34^.

## Strength and limitations

This study’s principal strength is its comprehensive investigation of a range of psychological variables, including premorbid psychological disorders, personality traits, somatization tendencies, and current levels of depression and anxiety. By integrating these factors, the study provides a nuanced understanding of how psychological characteristics are associated with dizziness severity in VS patients. The study identified several robust associations between psychological factors and different aspects of dizziness, which adds valuable insight into the complex relationship between these variables. For instance, the observed associations between conscientiousness, surgery-related changes in social support needs, and the severity of dizziness highlight the multifaceted nature of dizziness and its psychological impacts.

However, several limitations must be acknowledged. First, the cross-sectional design precludes causal inferences and does not capture the temporal dynamics between preoperative symptoms, psychological factors, and postoperative dizziness. Longitudinal studies are needed to determine whether psychological symptoms precede, accompany, or develop as a consequence of persistent vestibular symptoms. We did not assess duration and fluctuation of vestibular excitability, which was shown to have significant impact on dizziness handicap in vestibular migraine patients in previous studies^28^. Second, the absence of detailed neuro-otological evaluations precludes the identification of specific vestibular deficits or the degree of central compensation post-surgery. This limits the ability to attribute dizziness to psychological factors alone, as vestibular dysfunction and comorbid conditions may be equally relevant contributors. Third, the retrospective assessment of preoperative dizziness introduces potential recall bias, which may affect the accuracy and reliability of these self-reported baseline symptoms. This limitation is particularly relevant given the long postoperative interval in parts of the cohort (up to 36 years), as memory distortions and current symptom status may influence retrospective ratings. Such bias may lead to either over- or underestimation of preoperative symptom severity and should therefore be considered when interpreting associations involving preoperative dizziness. Accordingly, causal inferences regarding the temporal relationship between pre- and postoperative symptoms are limited. Fourth, we acknowledge that the study sample was recruited during routine clinical consultations and through an online survey distributed by a patient self-help organization. This combined recruitment strategy may have resulted in a non-representative sample because individuals involved in self-help organizations may be more likely to have higher symptom awareness, greater chronicity, or an increased psychological burden than the general VS population. Conversely, patients recruited in clinical settings may differ in terms of disease stage or treatment phase. Therefore, the present sample may not fully reflect the broader VS population, which limits the generalizability of the findings.

It is essential to evaluate the psychometric properties of the DHI, which, despite its widespread use, may exhibit suboptimal properties, as evidenced by extant research^35^. Despite the DHI’s limitations, it remains the most commonly utilized tool due to the lack of a more robust alternative in the existing literature. It is recommended that future research focus on validating and enhancing the current measurement tools, as well as investigating effective coping strategies and interventions to more effectively manage the impact of psychological factors on dizziness.

Finally, while this study identifies significant associations between psychological factors and dizziness, it does not examine specific coping mechanisms or rehabilitation interventions that may mitigate the effects of psychological distress. Further research should focus on identifying effective interventions tailored to patients with chronic vestibular symptoms, particularly those with a history of preoperative dizziness.

## Conclusion

This study identifies significant associations between psychological factors and dizziness severity in patients with VS following retrosigmoid microsurgery. Specifically, somatization, and anxiety were consistently associated with greater emotional and functional dizziness handicap. Higher scores on the GBI Social Support subscale were associated with greater dizziness handicap and independently predicted higher DHI scores. Because this subscale reflects surgery-related changes in the need for and reliance on social support rather than baseline social support resources, this finding may indicate that patients with greater postoperative disability experience greater changes in their support needs. Personality traits showed more heterogeneous associations, with conscientiousness demonstrating a positive relationship with dizziness handicap, potentially reflecting increased symptom monitoring, health awareness, or other individual differences in symptom perception rather than a direct effect on dizziness severity. These findings underscore the importance of considering psychological factors alongside vestibular variables in the assessment of persistent dizziness after VS surgery. However, given the cross-sectional design and lack of objective vestibular testing, no causal inferences can be drawn, and symptom mechanisms cannot be attributed to specific vestibular or functional diagnoses. Persistent dizziness in this population is likely multifactorial, potentially involving residual vestibular dysfunction, incomplete central compensation, and functional mechanisms. While symptom patterns may be compatible with PPPD, this framework cannot be confirmed in the present study and should be interpreted as a hypothesis requiring further clinical validation. Future research should therefore incorporate comprehensive neuro-otological assessments to better characterize vestibular contributions to persistent symptoms. Longitudinal study designs are also needed to clarify temporal relationships between preoperative symptom burden, psychological factors, and postoperative dizziness. From a clinical perspective, CBT may represent a promising approach for selected patients with persistent dizziness and relevant psychological comorbidities. Educational strategies addressing symptom interpretation, sensory processing, and maladaptive coping behaviors may further support recovery. Although complete symptom resolution may not always be achievable, meaningful reductions in symptom burden and improvements in quality of life represent realistic treatment goals. Finally, incorporating systematic assessment of psychological distress and premorbid vulnerability into routine clinical care may help identify patients at risk for persistent dizziness and guide early multidisciplinary intervention strategies.

## Data Availability

The datasets generated and/or analyzed during the current study are not publicly available due to the sensitive nature of the clinical and psychological data, but are available from the corresponding author on reasonable request.
